# Severe dysphagia due to an esophageal duplication cyst in sixth decade, unusual presentation of a rare pathology

**DOI:** 10.1186/s13019-023-02308-z

**Published:** 2023-07-26

**Authors:** Santiago A. Endara, Jaime R. Pinto, Gustavo A. Torres, Pablo A. Arias, M. Patricia Ponton, Gabriel A. Molina

**Affiliations:** 1 Department of Surgery Division of Cardiothoracic Surgery, Hospital Metropolitano, Av. Mariana de Jesus Oe 7/47 y Conclina, Edificio Diagnostico 2000 tercer piso 3/3, Quito, Ecuador; 2 Department of Surgery, Division of Cardiothoracic Surgery, Hospital Metropolitano, Quito, Ecuador; 3 Department of Internal Medicine, Division of Gastroenterology, Hospital de los Valles, Quito, Ecuador; 4 PGY1, General Surgery, Universidad Internacional del Ecuador (UIDE), Quito, Ecuador; 5 Department of Internal Medicine, Division of Pathology, Hospital Metropolitano, Quito, Ecuador; 6 Universidad San Francisco de Quito (USFQ), Quito, Ecuador

**Keywords:** Esophagus, Esophageal duplication cyst, Mediastinum

## Abstract

**Background:**

Esophageal duplication cysts are rare congenital tumors usually diagnosed and treated during childhood. Most of them are located in the mediastinum and appear as a mass besides the esophagus. Unfortunately, symptoms are non-specific and depend on the size and location of the mass; therefore, they can easily be missed. If symptoms appear, surgical resection is necessary to prevent troublesome complications.

**Case Presentation:**

We present the case of a 60-year-old woman who presented with severe progressive dysphagia and epigastric pain. After further evaluation, a paraesophageal cystic mass was found, and surgery was required. Non-communicating esophageal duplication cyst was the final diagnosis.

**Conclusion:**

Esophageal duplication cysts are a rare pathology in adults; their symptoms will vary depending on their size and location. Preoperative diagnosis is difficult as symptoms are non-specific and can be missed. If severe dysphagia, pain, or any other complication appears, surgery should not be delayed.

## Introduction

Esophageal duplication cyst is an unusual congenital disorder of the foregut; they are usually diagnosed during infancy and account for up to 10–15% of duplications of all foregut cysts [1, 2]. These cysts appear from a failure of vacuolization of the primitive esophagus and can produce symptoms due to esophageal and respiratory system compression, rupture, and infection [3, 4]. Surgery is required in symptomatic cases to prevent severe complications.

We present the case of a 60-year-old patient who presented severe dysphagia, weight loss, and epigastric pain due to a large esophageal duplication cyst. After surgery, she underwent complete recovery.

## Case Report

Patient is a 60-year-old woman without past medical history. She began to experience difficulty swallowing. The dysphagia was mild initially and did not affect her life, but as time passed, it worsened, first with solids and then with liquids. Due to this, she went to several physicians. Nonetheless, all endoscopies done at that time were normal; she grew tired of the doctors, and little by little, she changed her diet, she only ate liquids, and at one point, she couldn’t even swallow her own saliva.

As a result, she suffered weight loss and was brought to a community hospital by her family.

On clinical evaluation, an underweight patient (BMI 18.5) was encountered; she had mild epigastric pain; however, no tenderness was discovered; also, no masses or lymph nodes were found at that time.

Esophageal malignancy and pseudoachalasia were included in the differential, and complementary exams were needed.

**Fig. 1:**
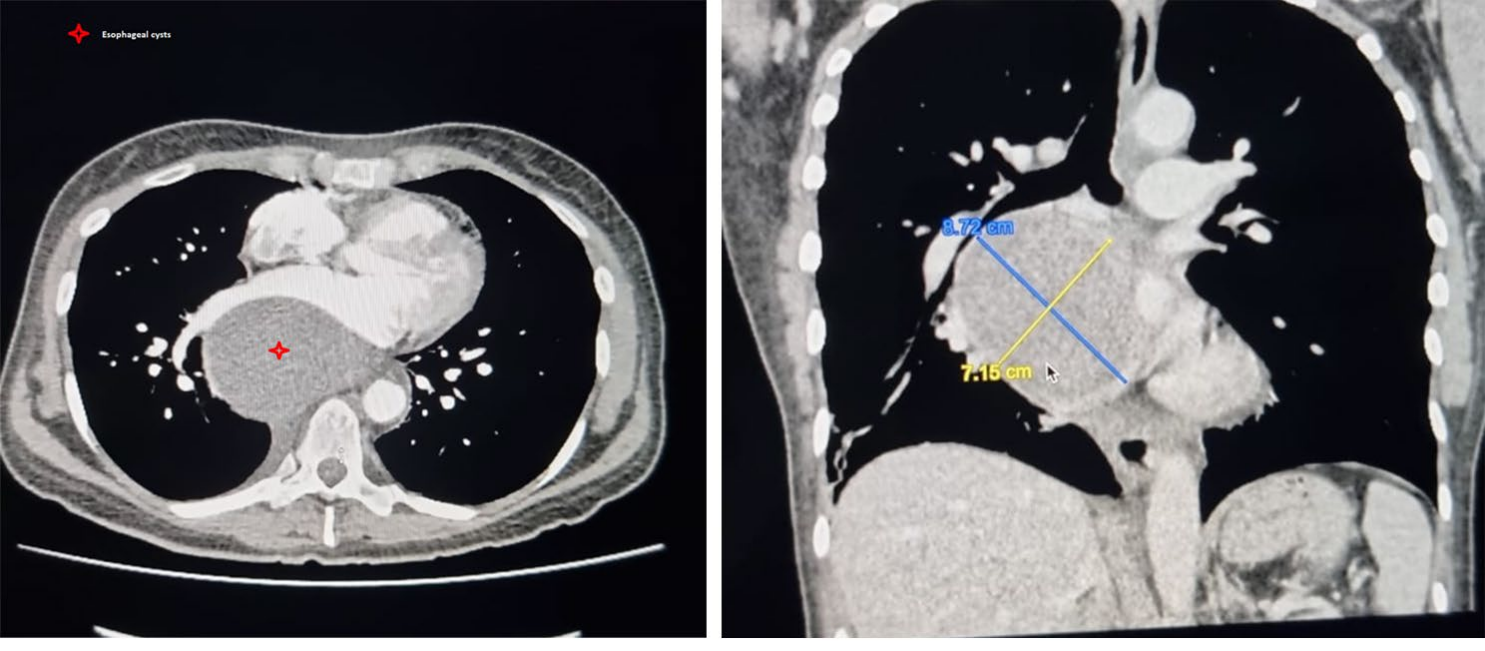
**A**: CT, The mass in the mediastinum besides the esophagus. **B**: CT, Cystic Mass displacing the organs

A new upper endoscopy was done; nonetheless, it didn’t reveal any pathology; therefore, a contrast-enhanced computed tomography was requested, revealing a 7,9 × 9,5 × 7,1 cm subcarinal mediastinal mass with multiple heterogeneous densities; the mass had round and regular edges, shifting the esophagus and trachea, and heart towards the left. As a result, a cardiothoracic assessment was needed, and the patient was transferred to our hospital. (Figs. 1 A, 1B). With these findings, benign and malignant tumors were among the differential, including; teratoma, bronchogenic cyst, and duplication cyst. Therefore, surgery was decided.

**Fig. 2:**
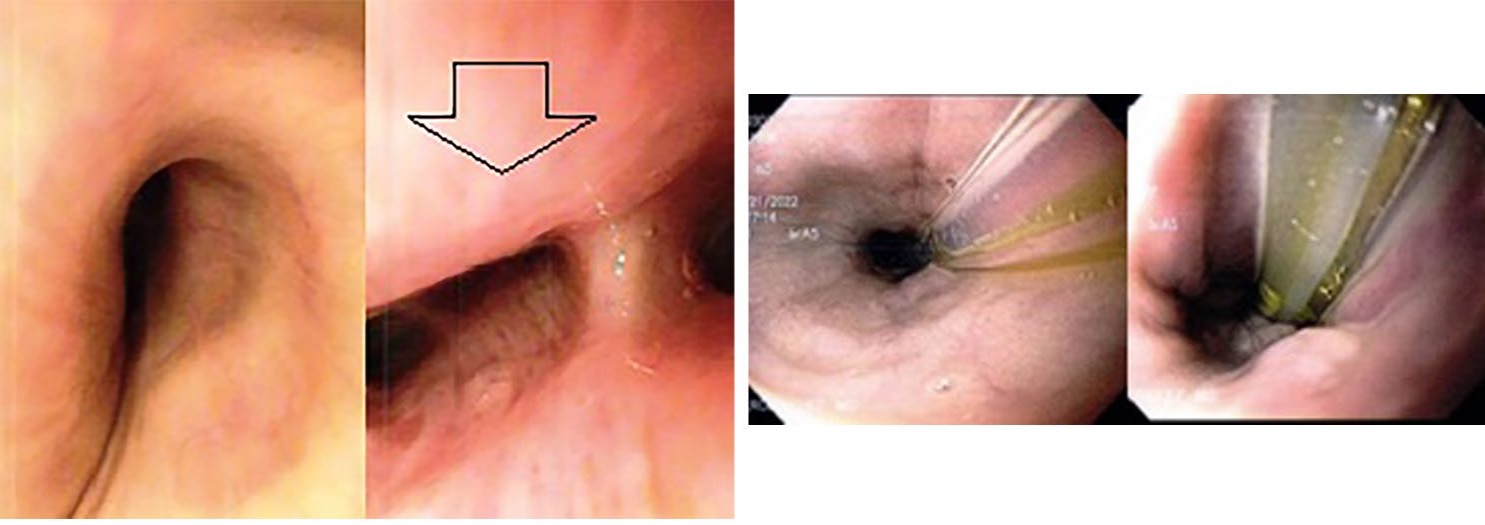
**A**: Transoperative bronchoscopy showing extrinsic compression of the upper segment of the right pulmonary lobe, arrow is showing the compression. **B**: Endoscopy, no compression is seen

After intubation, a bronchoscopy revealed a 30% extrinsic compression in the upper segment of the right lower pulmonary lobe (Fig. 2A), since endoscopic ultrasound wasn’t available at the time a right posterolateral thoracotomy was done. After retraction of the right lung, adequate exposure of the mediastinal mass was achieved. The mass had a thick whitish outer layer and was firmly attached to the carina, esophageal wall, and right lung. The mass was drained, obtaining 250 ml of whitish liquid, and was carefully dissected from surrounding structures.

**Fig. 3:**
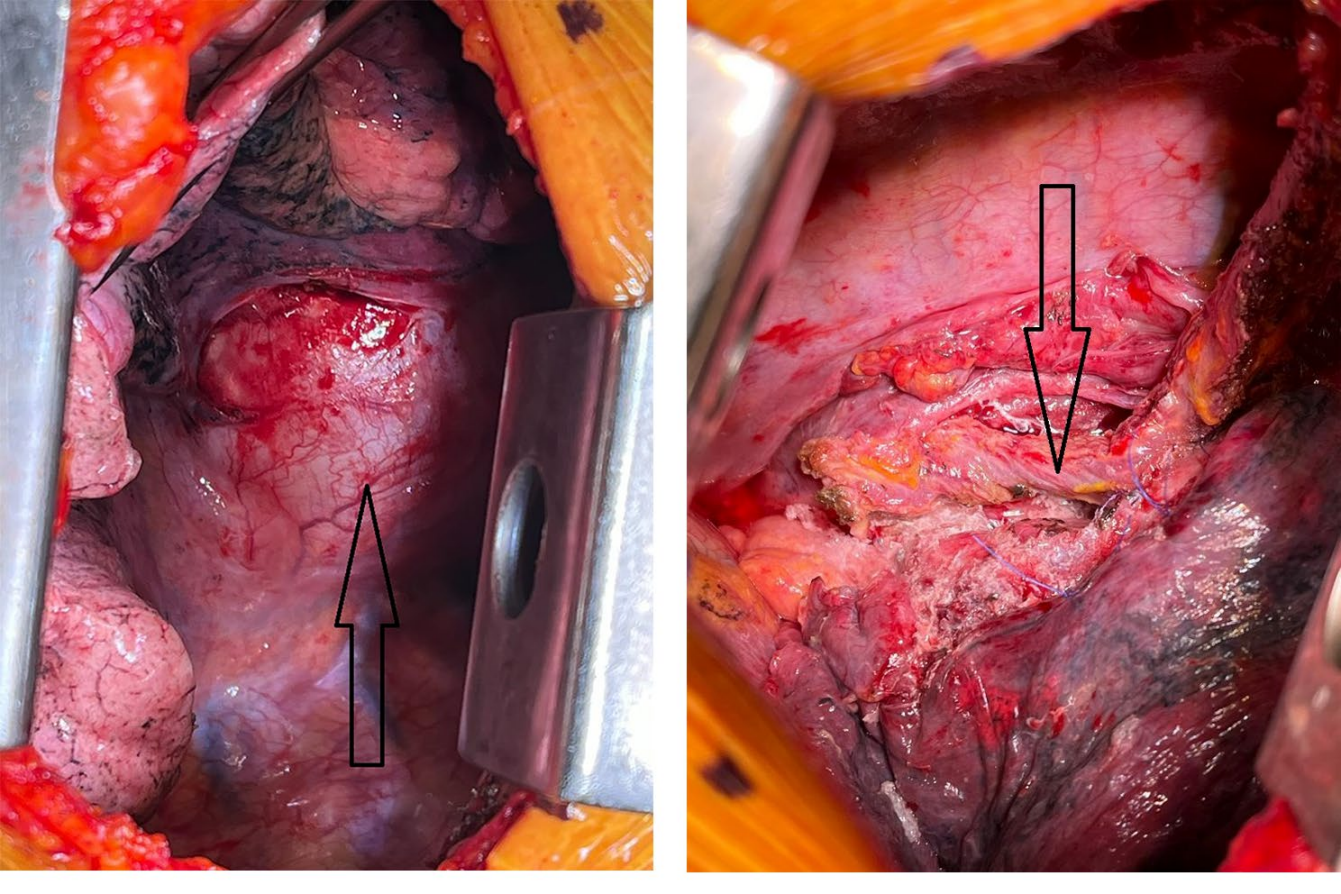
**A**: Mass attached to the esophagus, carina and lung; arrow is showing the mass. **B**: Complete resection of the mass, showing the esophagus on the arrow

Another endoscopy was completed prior to closure to ensure the integrity of the esophageal wall as the mass was attached to it. (Figure 3A and B)

Two chest tubes were placed, and the procedure was completed without complications.

**Fig. 4:**
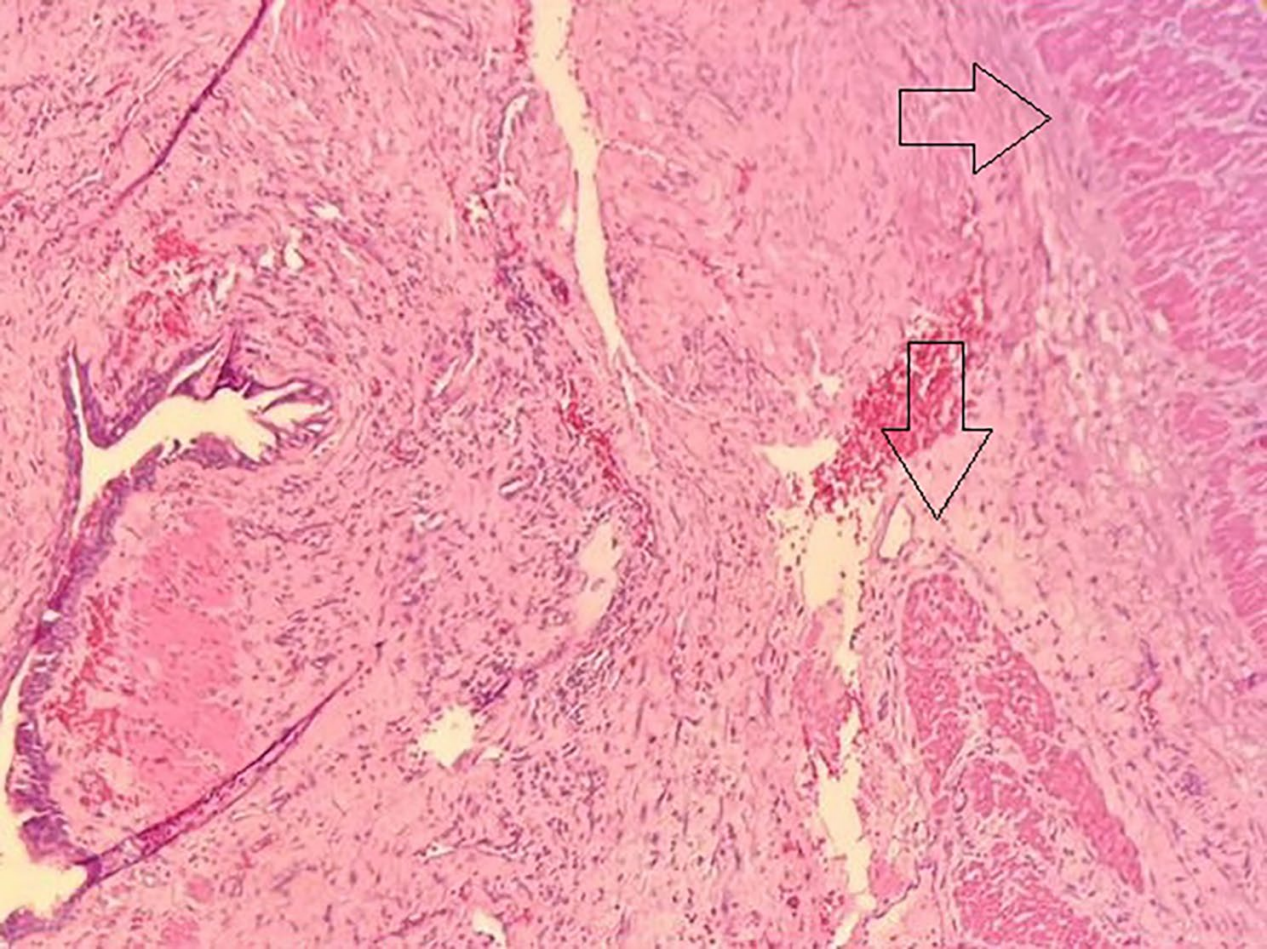
Pathology, mass wall enveloped by two muscular layers (shown on arrows) and lined by a pseudostratified columnar epithelium

Pathology reported a 6.5 × 5 × 2.5 cm mass; its wall had a thickness of 0.3 cm and was enveloped by two muscular layers. A pseudostratified columnar epithelium lined it without signs of malignancy. (Fig. 4) The final diagnosis was non-communicating esophageal duplication cyst. The culture of the fluid was negative, as no bacteria were found.

Her postoperative course was uneventful, the chest tubes were removed on the third postoperative day, and she was discharged on the seventh postoperative day without complications. On follow-ups underwent full recovery; she resumed her regular diet and 6 months after surgery she regained her normal weight.

## Discussion

Esophageal cysts are extremely rare congenital malformations [1, 2]. They were first described by Blasius et al. in 1711 and are the second most common esophageal tumors (1 in every 8200 live births) [1].

They tend to have a male predominance (2:1) and account for approximately 10% of all mediastinal tumors in children [2, 3]. Esophageal cysts can be categorized into simple and foregut cysts; the latter include the even rarer (0.5 a 2.5%) esophageal duplication cysts [1, 3]. Although the pathological pathways are still under research, esophageal duplication cysts are thought to appear from a failure of vacuolization of the primitive esophagus during the 4th to 8th week of life [3]. They may communicate with the esophagus or be isolated from it [4, 5]. They can also be associated with other congenital malformations, including cystic bronchiectasis, intrapulmonary bronchogenic cysts, and bronchial atresia [3, 6].

Esophageal duplication cysts are usually diagnosed (up to 80%) during infancy, and only a few patients (less than 150 in the English literature) remain asymptomatic until adulthood [3, 4]. A recent systematic review by Gonzalez-Urquijo et al., which pooled data from 97 adult patients with esophageal duplication cysts, found that most patients (81%) are symptomatic [7, 8]. As the cyst grows, it will cause symptoms depending on its involvement with surrounding structures [5, 6]. In our patient we believe this is what happened as the cyst grew the patient began to have more symptoms.

Pain, dysphagia, and reflux are the most common in adults, while airway obstruction and repeated pulmonary infection are more common in children [1, 4]. Adults can also lose weight due to the fear of experiencing dysphagia [6, 7, 9]. If the cyst is not promptly diagnosed, it might cause severe complications such as perforation, intramural hematoma, infection, and malignant transformation [5]. In our patient, the cyst grew in a way that affected the esophagus to the point that the patient was unable to eat; regretfully, she was misdiagnosed, leading to weight loss and severe dysphagia.

Esophageal duplications cysts share a common muscular wall with the native esophagus and are attached to it [2, 10]. Two smooth muscle layers surround the cysts; this is essential to differentiate them from bronchogenic cysts that contain cartilage in their wall [4]. They can be lined by squamous, cuboid, columnar, or ciliated columnar epithelium. (1) [1, 3] Ectopic GI mucosa of the esophagus can confuse the diagnosis as it can mimic many gastrointestinal pathologies, especially those involving the ileum, such as Meckel diverticulum [2, 11].

Esophageal duplications cysts tend to appear in the right posterior-inferior mediastinum or lower-middle esophagus [1, 2]. As it was found in our patient.

Preoperative diagnosis needs several imaging methods like CT or MRI [1, 7]. Nonetheless, endoscopic ultrasonography is the best available tool since it can easily differentiate between a solid and a liquid mass and specify which layer of the esophagus is compromised [1, 2, 12]. Other studies like barium swallow can aid in the diagnosis to determine the precise location of the lesion and its relationship with the esophageal hiatus [7, 8]. Still, it won’t be able to distinguish between a duplication cyst and a leiomyoma. A Tc-99 m scan can also be helpful if the gastric epithelium is present [4, 8]. In our case, as the endoscopic ultrasound wasn’t available, a CT, bronchoscopy, and endoscopy were done to reach a final diagnosis.

Observation may be possible in asymptomatic patients, especially in patients at high surgical risk; nonetheless, complete resection is mandatory if symptoms are present [1, 2, 13]. Open, endoscopic, and thoracoscopic approaches are available. Nevertheless, extreme care must be considered when dissecting the cyst and the esophagus to prevent perforation [1, 9, 14]. As it was done with our patient.

Esophageal duplication cysts are still rare in adults and are frequently located in the distal esophagus; since larger cysts are more likely to cause symptoms, we should always keep this pathology among the differential diagnoses to achieve an accurate diagnosis and avoid cases such as our patient’s. Interestingly, this case also proves that the doctor–patient relationship is essential; if we can connect with our patients and investigate more about their pain instead of just reviewing exams, we can improve our practice and get to know the patients better.

## Conclusion

This report shows a case of an esophageal duplication cyst found in adulthood; the dysphagia and abdominal pain were progressive and eventually impaired the patient’s life. Although there are cases in which esophageal duplication cysts may go unnoticed, dysphagia is a symptom that should always be investigated to prevent events such as this. Esophageal duplication cysts are rare; however, this case proves that they can grow and produce serious complications, ones that should never go unnoticed.

## Data Availability

All data presented in this manuscript is made by the authors and can be accessed by the Editor on demand.

## References

[1] Parshin V, Osminin S, Komarov R, Vetshev S, Strakhov Y, Ivashov I (2021). Rare diseases of esophagus: Surgical treatment of cysts in adults. Case report. Int J Surg case Rep.

[2] Chernousov KFS, Bogopolsky AF, Manual for Physicians PM. 2000. Esophageal surgery. Manual for physicians, esophageal surgery. — M.: Moscow Publishers, 2000. – 352 p., ill., Moscow.

[3] Wootton-gorges SL, Eckel GM, Poulos ND, Milstein JM. 2002. Duplication of the cervical esophagus: a Case Report and Review of the Literature; pp. 533–5. 10.1007/s00247-002-0693-8 12107589

[4] Carter B, Benveniste M, Madan R, Godoy M, de Groot P, Truong M (2017). ITMIG classification of Mediastinal Compartments and Multidisciplinary Approach to Mediastinal Masses. Radiographics.

[5] Wiechowska-Kozłowska A, Wunsch E, Majewski M, Milkiewicz P (2012). Esophageal duplication cysts: endosonographic findings in asymptomatic patients. World J Gastroenterol.

[6] Obasi PC, Hebra A, Varela JC (2011). Excision of esophageal duplication cysts with robotic-assisted thoracoscopic surgery. JSLS J Soc Laparoendosc Surg.

[7] Ringley C, Bochkarev V, Oleynikov D (2006). Esophageal duplication cyst–a guest case in robotic and computer-assisted surgery from the University of Nebraska Medical Center. MedGenMed: Medscape general medicine.

[8] Gonzalez-Urquijo M, Hinojosa- Gonzalez DE, Padilla-Armendariz DP (2022). Esophageal duplication cysts in 97 adult patients: a systematic review. World J Surg.

[9] Pisello F, Geraci G, Arnone E, Sciuto A, Modica G, Sciumè C (2009). Acute onset of esophageal duplication cyst in adult. Case report. Il Giornale di chirurgia.

[10] Pogliani L, Zanfrini E, Tabacco D, Meacci E, Margaritora S, Nachira D, Porziella V. Esophageal duplication cyst recurrence: case report and literature review. Annals Of Esophagus. 2021;5. 10.21037/aoe-2020-26.

[11] Adler D, Liu R (2014). Duplication cysts: diagnosis, management, and the role of endoscopic ultrasound. Endoscopic Ultrasound.

[12] Wahi J, Safdie F (2023). Esophageal duplication cysts: a clinical practice review. Mediastinum.

[13] Zhang Z, Jin F, Wu H, Tan S, Tian Z, Cui Y. Double esophageal duplication cysts, with ectopic gastric mucosa: a case report. J Cardiothorac Surg. 2013;8(1). 10.1186/1749-8090-8-221. 10.1186/1749-8090-8-221 PMC4222059 24289795

[14] Takemura M, Yoshida K, Morimura K. Thoracoscopic resection of thoracic esophageal duplication cyst containing ectopic pancreatic tissue in adult. J Cardiothorac Surg. 2011;6(1). 10.1186/1749-8090-6-118. 10.1186/1749-8090-6-118 PMC3189109 21943206

